# Genetic Testing for CYP450 Polymorphisms to Predict Response to Clopidogrel: current evidence and test availability: Application: Pharmacogenomics

**DOI:** 10.1371/currents.RRN1180

**Published:** 2010-09-20

**Authors:** Renée M. Ned

**Affiliations:** Office of Public Health Genomics, Centers for Disease Control and Prevention

## Abstract

The anti-platelet agent clopidogrel bisulfate (sold under the trade name Plavix in the United States) is a widely prescribed medication for the prevention of blood clots in patients with acute coronary syndrome, in those who have suffered other cardiovascular disease-related events such as ischemic stroke, and in patients who are undergoing percutaneous coronary intervention. Response to clopidogrel varies substantially due to genetic and acquired factors. Patients who experience recurrent cardiovascular ischemic or thrombotic events while taking clopidogrel are typically described as non-responsive or resistant.

The drug's oxidation is mainly dependent on the cytochrome P450 enzyme 2C19 (CYP2C19). Patients with certain genetic variants in CYP2C19 have been found to have lower levels of the active metabolite, less platelet inhibition, and greater risk of major adverse cardiovascular events such as heart attack, stroke, and death. Testing for CYP2C19 polymorphisms may identify patients who will not respond adequately to the standard clopidogrel regimen and who should, consequently, be given an alternate treatment strategy. This article outlines the evidence concerning pharmacogenetic testing for clopidogrel response, including data on clinical validity and clinical utility, and summarizes the currently available tests marketed for this purpose.

## **Clinical Scenario**

Pharmacogenetic testing to identify patients at risk of an inadequate response to the standard clopidogrel regimen so that alternate treatment strategies can be initiated, with the goal of preventing adverse cardiovascular events such as stent thromboses, recurrent ischemic events, and death.

**Test Description**

Genetic polymorphisms in several genes (e.g., *CYP1A2*, *CYP3A4*, and *CYP3A5*) have been studied for an association with antiplatelet response and clinical outcomes in those taking clopidogrel. However, despite the many enzymes known to be involved in the metabolism of clopidogrel, only genetic variation in *CYP2C19* has been consistently and significantly associated with clopidogrel response in multiple populations [1] [2] [3].

The numerous commercial pharmacogenetic tests that are available genotype variants in *CYP2C19* for the purpose of predicting response to clopidogrel (see Table 1). These tests differ in genotyping methodology, sample type required, and availability (direct-to-consumer or physician-ordered). However, all tests include, at a minimum, the most common alleles (*1, and loss-of-function alleles *2 and *3), which have been shown to account for most of the variability in response to clopidogrel [1] [2]. Some tests also include other identified reduced-function variants (named *4, *5, *6, *7, *8, *9, and *10). A newer allele, CYP2C19*17, has been described that is associated with increased enzymatic activity and ultra-rapid drug metabolism [4] [5], which in turn is predicted to result in higher levels of the active metabolite of clopidogrel.

Each test includes: 

- Analysis of multiple single nucleotide polymorphisms in *CYP2C19*.
- Genotype-based prediction of CYP2C19 enzymatic activity to categorize patients as:

- Extensive metabolizers [carrying two "normal" alleles (i.e., *1/*1)].
- Intermediate metabolizers [carrying one reduced-function allele (e.g., *1/*2)].
- Poor metabolizers [carrying two reduced-function alleles (e.g., *2/*2 or *2/*3)].
- Ultra-rapid metabolizers [carrying one or two increased-function alleles (i.e., *1/*17 or *17/*17), though it is not clear whether *1/*17 carriers have a different phenotype than *1/*1 carriers].

**Table 1.  Pharmacogenetic Tests for Clopidogrel Response**

**Table:** 

**Test**	**Company**	***CYP2C19 *** **Variants Included**	**Sample Type**	**Available Direct-to-consumer (DTC)**
AccuType™ CP testing for Clopidogrel CYP2C19 Genotype (*1, *2, *3, *4, *5)	Quest Diagnostics	*1, *2, *3, *4, *5	Whole blood or saliva	No
Clopidogrel P450 Genotype	Quest Diagnostics	*1, *2, *3 (also includes 26 variants in *CYP2D6*)	Whole blood	No
Plavitest for Clopidogrel Resistance (CYP 2C19)	Genelex Corporation	*1, *2, *3, *4, *5, *6, *7, *8	Whole blood or buccal (cheek) swab	No
Comprehensive DNA Drug Sensitivity Test	Genelex Corporation	Unspecified	Whole blood or buccal (cheek) swab	No
Medications Panel [part of the “Health Compass”]	Navigenics, Inc.	“Two of the most common genetic variants in *CYP2C19*”	Saliva	No
Clopidogrel Efficacy [part of the “Health Edition” test]	23andMe	*1, *2, *3, *4, *8, and *17	Saliva	Yes
Drug Response (Medication) [part of the “Total Health Insight” test]	Pathway Genomics	Unspecified	Saliva	Yes, except in New York state
Clopidogrel Activation	Matrix Genomics	*1, *2, *3, *4, *5, *6, *7, *8, *9, *10, *17	Buccal (cheek) swab	Yes
Cytochrome P450 2C19 *(CYP2C19)* 7 Mutations	ARUP Laboratories	*1, *2, *3, *4, *5, *6, *7, *8	Whole blood	No
CYP450 2C19 Gene Test / Genetic Physician Consult	MyMedLab, Inc.	Unspecified	Buccal (cheek) swab	Yes, though orders must be approved by a MyMedLab physician
Clopidogrel Genetic Test	TheranostiCs Lab	Unspecified	Buccal (cheek) swab	Yes

Note:  These tests were found through Google searches combining terms such as “clopidogrel”, “genetic test” and “CYP2C19” and by searching individual websites of known commercial genetics companies (such as Pathway Genomics).  Attempts were made to make this table comprehensive. However, there are tests that genotype variants in *CYP2C19* that either do not specify that the test is for pharmacogenetic purposes, or do not specifically mention that the test can be used in determining response to clopidogrel. Such tests have not been included in this table.

**Disclaimer:**

**Inclusion of tests in this table does not constitute an endorsement of any test by the Centers for Disease Control and Prevention (CDC) nor the Department of Health and Human Services (DHHS) of the U.S. government. No endorsement should be inferred.**

## **Public Health Importance**

Clopidogrel is the second highest top-selling drug in the world [6]; approximately 29 million prescriptions were dispensed in 2008 in the United States alone [7]. 

Estimates of the prevalence of laboratory-defined clopidogrel non-responsiveness vary widely, but have been estimated at 21-26% overall [8] [9] [10].

An inadequate response to clopidogrel can cause stent thromboses, recurrent ischemic events, and death [8] [9] [10]. Approximately 9% of patients taking clopidogrel have a major adverse cardiovascular event such as myocardial infarction, stroke, or cardiovascular death [11] [12]. Alternately, an enhanced response to clopidogrel may cause major bleeding [13], which typically occurs in ≈1.5% of patients (though much higher rates have been reported) [11] [12] [13] [14]. 

Sizeable proportions of some populations possess at least one loss-of-function *CYP2C19* allele (typically *2 or *3) that could affect clopidogrel response: ≈ 30-50% of Asians, 11-16% of Caucasians, and 14-25% of African-Americans [1] [15] [16]. It is estimated that the *CYP2C19* "poor metabolizer" phenotype is exhibited by 10-25% of Asians, 2-3% of Caucasians, and 4% of African-Americans [1] [15] [16]. CYP2C19*2 and *3 account for more than 95% of cases of the "poor metabolizer" phenotype [1]. The allele frequency of CYP2C19*17 is estimated as 18-27% among Caucasians, 17-18% among Africans/African-Americans, and 0.5 – 4% among Asians [4]

Recently, the U.S. Food and Drug Administration (FDA) issued a black box warning for clopidogrel due to the reduced effectiveness of the drug in poor metabolizers [17] [18].

## **Published Reviews, Recommendations and Guidelines** 

**Systematic evidence reviews**

None.

**Recommendations by independent group**

None.

**Guidelines by professional groups**

In July 2010, the American College of Cardiology Foundation (ACCF) and the American Heart Association (AHA) published a Clinical Alert in response to the FDA's black box warning on clopidogrel [19].  The report stated that:

- The evidence base is insufficient to recommend routine genetic testing at the present time.
- "Clinical judgment is required to assess clinical risk and variability in patients considered to be at increased risk. Genetic testing to determine if a patient is predisposed to poor clopidogrel metabolism ("poor metabolizers") may be considered before starting clopidogrel therapy in patients believed to be at moderate or high risk for poor outcomes. This might include, among others, patients undergoing elective high-risk PCI procedures (e.g., treatment of extensive and/or very complex disease)." 

**Other guidelines** 

The U.S. Food and Drug Administration (FDA) determined in 2009 that the available data have “provided compelling evidence that genetic variation in *CYP2C19* is a significant and independent predictor of clopidogrel pharmacokinetics, pharmacodynamics and clinical response”, which prompted the FDA to change clopidogrel’s prescribing information [3] [20] [21]. More recently (March 12, 2010), the FDA issued a black box warning for clopidogrel [17] [18] due to the reduced effectiveness of the drug in poor metabolizers. The FDA recommended that health professionals be aware that some patients may be poor metabolizers of clopidogrel because of low CYP2C19 activity and to also "be aware that tests are available to determine patients' CYP2C19 status" [17]. **The FDA neither mandated nor explicitly recommended *CYP2C19* genetic testing in patients prescribed clopidogrel**, and the agency did not offer any specific guidance on drug dosing in *CYP2C19* variant allele carriers [17]. The November 2009 label update did recommend that doctors avoid use of clopidogrel "in patients with impaired CYP2C19 function due to known genetic variation or due to drugs that inhibit CYP2C19 activity" [21], while the more recent warning no longer specifically advises against use of clopidogrel in *CYP2C19* poor metabolizers [17].

## **Evidence Overview** 

***Analytic Validity*:**Test accuracy and reliability in identifying *CYP2C19* genotypes (analytic sensitivity and specificity).

- When reported, accuracy of the various genotyping methods/platforms used in commercial tests is noted as ≥ 99% [22] [23] [24] [25] [26] [27]. However, no information on analytic sensitivity or specificity can be found for the tests offered by Pathway Genomics [28], Matrix Genomics [29], or MyMedLab [30], or for some tests offered by Quest Diagnostics [31] or Genelex Corporation [32].
- Cross validation of the techniques used in *CYP2C19 *genotyping assays, along with their reliability, specificity, and reproducibility, is extremely limited [19].

***Clinical Validity*:**Test accuracy and reliability in predicting response to clopidogrel (predictive value).

- Numerous published studies have examined the relationship of *CYP2C19* alleles to clopidogrel response [33].

            HuGE Navigator: query "clopidogrel and CYP2C19"

- At least 22 studies have examined pharmacokinetic and/or pharmacodynamic responses by *CYP2C19 *genotype; these studies differed in the populations tested and the specific pharmacokinetic or pharmacodynamic measure(s) that were employed (most studies reviewed in [1] [34]). Twenty studies examined reduced-function variants (mostly *2 alone or in combination with other variants) [2] [35] [36] [37] [38] [39] [40] [41] [42] [43] [44] [45] [46] [47] [48] [49] [50] [51] [52] [53]. All studies except one [37] reported statistically significant differences in clopidogrel response in reduced-function allele carriers. In these 19 studies, p values ranged from 4.3x10^-11^ to <0.05.
- Seven studies have examined the association of the *17 variant with pharmacokinetic or pharmacodynamic responses specifically to clopidogrel, with inconsistent results [2] [39] [44] [48] [53] [54] [55]. Only one of these studies measured plasma levels of the clopidogrel active metabolite. However, *17 allele carriers were grouped with *1/*1 extensive metabolizers; and so individual effects of the *17 allele on clopidogrel metabolism cannot be determined [44].
- Only one clinical trial explicitly reported measures of clinical validity. In the EXCELSIOR (Impact of Extent of Clopidogrel-Induced Platelet Inhibition During Elective Stent Implantation on Clinical Event Rate) trial, the sensitivity and specificity of CYP2C19*2 carrier status for detecting high on-clopidogrel residual platelet aggregation (RPA) was 45.1% and 75.0%, respectively [35].^  ^Measures of sensitivity and specificity can be calculated from data reported in some of the other published studies.
- No investigators explicitly included estimates of positive predictive value (PPV) or negative predictive value (NPV) for clopidogrel response in their original published reports. Using data from the EXCELSIOR report [35], estimates of PPV and NPV are 41.6% and 77.6%, respectively.

***Clinical Utility*: **Net benefit of test in improving health outcomes.

- A recent meta-analysis of seven prospective cohort studies was published that examined the CYP2C19*2 variant and cardiovascular recurrences in 8,043 coronary artery disease patients taking clopidogrel. The authors found a statistically significant increased risk of major adverse cardiovascular events in CYP2C19*2 carriers (RR = 1.96) and an even greater risk when stent thrombosis was analyzed separately using four studies that included 4,975 patients (RR = 3.82) [56].
- A more recent meta-analysis that was not restricted to prospective cohort studies examined the CYP2C19*2 variant and the incidence of major adverse cardiovascular events (MACE) and mortality in patients taking clopidogrel [57]. The pooled data showed that CYP2C19*2 carriers had a statistically significant increased risk of MACE (OR = 1.29) using data from ten studies (11,959 patients). Carriers of *2 also had statistically significant increases in risk for stent thrombosis (OR = 3.45 using data from 4 studies including 4,905 patients) and death (OR = 1.79 using data from 5 studies including 6,225 patients). Four studies including 5,694 patients allowed the separate assessment of heterozygotes and homozygotes of the *2 allele. Increased risks for MACE and stent thrombosis were noted for both groups, though the odds ratio for MACE was significant only for homozygotes [57].
- Hulot and Fuster [58] calculated the PPV for clinical outcomes of *CYP2C19* loss-of-function variants using data from two studies. They estimate the PPV for a cardiovascular event is between 12% [36] and 20% [59].
- Most studies have examined *CYP2C19* genotypes only among patients taking clopidogrel. In the only published study to date that included a placebo-controlled group, neither CYP2C19*2 or *3 allele carriage nor metabolizer phenotype influenced the effect of clopidogrel on cardiovascular disease-related outcomes compared to the placebo group [60].
- Tests that assay CYP2C19*17 may also identify individuals at increased risk for bleeding events [13] [14] or those who may derive a larger benefit (reduction in cardiovascular events) with clopidogrel treatment compared to placebo [60].
- There are no published clinical trial data evaluating the net benefit of *CYP2C19* testing prior to administration of clopidogrel in improving health outcomes.
- It is unknown if the risk from a given individual's genomic profile changes over time, depending on the specific clinical scenario (for example, acute coronary syndrome versus stable angina pectoris) [19]. However, a meta-analysis demonstrated that the increased occurrence of major adverse cardiovascular events and of mortality in CYP2C19*2 carriers was independent of patients' baseline cardiovascular risk [57].
- Any benefits of DNA testing may be hampered by the following considerations:
  - The response to clopidogrel is unclear for some *CYP2C19* genotypes, such as reduced-function alleles in the presence of *17. The only study to directly assess the combined effect of *17 and reduced-function alleles (in this case, *2) found a gradient of effect on pharmacodynamic response to clopidogrel [53].
  - Genotyping only for *CYP2C19* alleles does not capture all of the genetic variability in pharmacodynamic, pharmacokinetic, or clinical responses to clopidogrel. Variants in the *ABCB1* gene also seem to be potentially important to the interindividual variability in these phenotypes [3] [14] [61] [62].
  - Currently, there are no standardized clinical guidelines to manage patients with an inadequate response to clopidogrel. Typically, one or more alternatives are tried: higher loading or maintenance doses of clopidogrel, dual therapy with aspirin if this has not already been initiated, or treatment with another antiplatelet medication [19].

##  **Links**

- Human Cytochrome P450 (CYP) Allele Nomenclature Committee, CYP2C19 allele nomenclature (http://www.cypalleles.ki.se/cyp2c19.htm), last accessed: March 17, 2010.
- **HuGE Navigator** **:** query "clopidogrel and CYP2C19"
- **U.S. Food and Drug Administration:** Table of Valid Genomic Biomarkers in the Context of Approved Drug Labels
- **ClinicalTrials.gov:** Clopidogrel and CYP2C19. Note: not all clinical trials list a pharmacogenetic component to their study, even if one exists.  For instance, some studies have been found to include genetic analyses only after publication of those data. Consequently, searching for "clopidogrel and CYP2C19" or a similar search will not return all relevant studies. 
- **PharmGKB** **:** clopidogrel, clinical PGx for clopidogrel

Last updated: *September 16, 2010*

## **Acknowledgments**

I would like to thank Shelley Reyes, Nicole F. Dowling, and Cecelia Bellcross in the Office of Public Health Genomics for comments and guidance. 

## **Funding information**

This work was supported by the Office of Public Health Genomics, Office of Surveillance, Epidemiology, and Laboratory Services, Centers for Disease Control and Prevention.

## **Competing interests**

The author declares that no competing interests exist.

### **Disclaimer**

The CDC does not offer medical advice to individuals. If you have specific concerns about your health or genetic testing, we suggest that you discuss them with your health care provider.
